# The Role of Myrrh Metabolites in Cancer, Inflammation, and Wound Healing: Prospects for a Multi-Targeted Drug Therapy

**DOI:** 10.3390/ph15080944

**Published:** 2022-07-29

**Authors:** Rasha Saad Suliman, Sahar Saleh Alghamdi, Rizwan Ali, Dimah Aljatli, Norah Abdulaziz Aljammaz, Sarah Huwaizi, Rania Suliman, Khawla Mohammed Kahtani, Ghadeer M. Albadrani, Tlili Barhoumi, Abdulelah Altolayyan, Ishrat Rahman

**Affiliations:** 1 Department of Pharmacy, Fatima College of Health Sciences, Abu Dhabi 3798, United Arab Emirates; 2 College of Pharmacy, King Saud Bin Abdulaziz University for Health Sciences, Riyadh 11461, Saudi Arabia; aljatli097@ksau-hs.edu.sa (D.A.); aljammaz369@ksau-hs.edu.sa (N.A.A.); kahtani085@ksau-hs.edu.sa (K.M.K.); barhoumitl@mngha.med.sa (T.B.); 3 Medical Research Core Facility and Platforms, King Abdullah International Medical Research Center (KAIMRC), Ministry of National Guard Health Affairs, Riyadh 11481, Saudi Arabia; aliri@ngha.med.sa (R.A.); huwaizisa@ngha.md.sa (S.H.); altolayyanab@ngha.med.sa (A.A.); 4 Clinical Laboratory Sciences, Prince Sultan Military College of Health Sciences, Dahran 31448, Saudi Arabia; rsuliman@psmchs.edu.sa.sa; 5 Department of Biology, College of Science, Princess Nourah bint Abdulrahman University, Riyadh 11671, Saudi Arabia; gmalbadrani@pnu.edu.sa; 6 Department of Basic Dental Sciences, College of Dentistry, Princess Nourah Bint Abdulrahman University, Riyadh 40886, Saudi Arabia; imrahman@pnu.edu.sa

**Keywords:** myrrh resin methanolic extract 1, in vivo anti-inflammatory 2, computational activity predictions 3, natural products 4, leukemia 5, breast cancer 6, ADME 7, target prediction 8

## Abstract

Background: Myrrh extract is a well-known medicinal plant with significant therapeutic benefits attributed to the activity of its diverse metabolites. It has promising activity against cancer and inflammatory diseases, and could serve as a potential therapeutic alternative since most therapeutic agents have severe side effects that impair quality of life. Method: The current study identified the active metabolites from the myrrh resin methanolic extract. Then, the extracts were tested for in vitro anti-inflammatory and anti-cancer activity using cancer cell lines and Tamm-Horsfall Protein 1 (Thp-1)-like macrophage cell lines. Furthermore, using an in vivo rat model, the extracts’ anti-inflammatory and wound-healing activity was investigated. In addition, in silico predictions of the myrrh constituents highlighted the pharmacokinetic properties, molecular targets, and safety profile, including cytochrome P 450 (CYP) inhibition and organ toxicity. Results: Nine secondary metabolites were identified, and computational predictions suggested a good absorption profile, anticancer, anti-inflammatory, and wound-healing effects. The myrrh extract had moderate cytotoxic activity against both HL60 and K562 leukemia cell lines and the KAIMRC1 breast cancer cell line. Myrrh caused a dose-dependent effect on macrophages to increase the reactive oxygen species (ROS) levels, promote their polarization to classically activated macrophages (M1) and alternatively activated macrophages (M2) phenotypes, and consequently induce apoptosis, highlighting its ability to modulate macrophage function, which could potentially aid in several desired therapeutic processes, including the resolution of inflammation, and autophagy which is an important aspect to consider in cancer treatment. The topical application of myrrh improved wound healing, with no delayed inflammatory response, and promoted complete re-epithelization of the skin, similar to the positive control. In conclusion, we provide evidence for the methanolic extract of myrrh having cytotoxic activity against cancer cells and anti-inflammatory wound-healing properties, which may be attributed to its role in modulating macrophage function. Furthermore, we suggest the active constituents responsible for these properties, which warrants further studies focusing on the precise roles of the active metabolites.

## 1. Introduction

Recent trends in return to natural alternative medicines have led to the spread of herbal studies to treat many diseases. Myrrh has a long history of widespread traditional use in most Eastern societies as a food additive and fragrance used in perfume and incense. It is also well known for its extensive health benefits to heal wounds, combat pain, inflammatory conditions, e.g., arthritis and obesity, and to treat infections, including leprosy and syphilis [1]. Originating from Arabia, myrrh is a yellow, fragrant oleo gum resin that belongs to the *Burseraceae* family and is secreted from the bark of the genus *Commiphora* tree [2]. The safe use of myrrh as a food additive was approved by the Food and Drug Administration (FDA) in 1992 and, as such, is included in the generally recognized as safe (GRAS) substances list and included in the plant and part list by the Council of Europe [3,4].

In the past decades, several researchers have sought to demonstrate and prove the efficacy of myrrh as an antioxidant, anti-microbial, and anti-cancer agent. The highly desirabe pharmacological properties of myrrh are attributed to the constituents and diverse classes of phytochemicals and metabolites found in the gum. Hundreds of organic compounds, many minerals, and other inorganic compounds, such as calcium, magnesium, aluminum, and phosphorus, have been identified [5]. Myrrh is reported to contain a small percentage (2–8%) of volatile oil fraction (myrrhol), a 23–40% resin fraction (myrrhin) making up an ether soluble and insoluble fraction, and a 40–60% gum proportion constituting mainly protein and carbohydrates [6]. The volatile oil contains eugenol [7], cumin aldehyde, monoterpenes, diterpenes, sesquiterpenes, lindestrene, and furanoeudesma-1,3-diene [8]; the latter two are responsible for the distinct aroma and analgesic benefits of myrrh [9]. Myrhhin or the resin fraction contains α-, β- and γ-commiphoric acid, α- and β-heerabomyrrhol, and other phenolic compounds such as protocatechuic acid and pyrocathechin [10]. The ether extract of the resin containing Commiphora was an effective antibacterial in wound infections [11]. Myrrh is a promising compound for treating ulcerative colitis, a chronic inflammatory condition. Myrrh treatment attenuated oxidative and inflammatory processes in rats with acetic acid-induced ulcerative colitis by lowering the levels of inflammatory mediators [12]. Myrrh’s metabolites induced cytotoxic activity against human prostate cancer PC3 [13,14] and furano-sesquiterpenoids isolated from Arabic *Commiphora Myrrh* resin induced apoptosis of human hepatocellular carcinoma HepG2 cells with a half-maximal inhibitory concentration (IC_50_) of 3.6 μM [15,16]. Indeed, numerous studies in vitro and in vivo show that myrrh and its metabolites are antimicrobial and have cytotoxic properties against several different cancer cells [17,18,19].

Cancer is an increasingly common disease. Dysregulated tissue growth, leading to invasion and metastasis, impairs normal body function and can lead to death [20]. Despite the tremendous progress in cancer management, the number of estimated cancer-related deaths in the year 2020 was 9,958,133 worldwide [21]. While the latest advancements in therapy have led to considerable strides in approaching and combatting cancer, the side effects of most of the therapeutic options threaten human health and significantly disturbing the patients’ quality of life. Therefore, the use of natural products and their naturally occurring metabolites in treating cancers may achieve the desired cytotoxic effects on cancer cells with no significant damage to the surrounding healthy tissues, thus displaying selectivity to diseased cells and potentially overcoming the limitations of other treatments. In addition, inflammatory diseases and infectious diseases seriously threaten human health, causing a rapid increase in mortality rate [8,22,23]. Although the continued discovery of synthetic compounds has led to progress in reducing mortality and increasing patients’ quality of life, most FDA-approved drugs, such as non-steroidal anti-inflammatory drugs (NSAIDs), corticosteroids, and other immunosuppressants, have serious adverse effects [24,25,26,27,28]. Furthermore, the rampant use of antimicrobials has led to superbugs with resistance to many known drugs. Thus, there is a need to identify natural alternatives that would be advantageous in treating infections and resolving pro-inflammatory conditions.

Macrophages are fundamental to the function of the innate immune system as part of the immediate response to stress, dysfunction, and disease. Hence, they are crucial in regulating inflammatory responses. Macrophages also contribute to the effector phase of the adaptive immune responses, being foremost to present antigens, thus initiating the immune response [29,30,31,32,33]. The classical phenotypes are M1 pro-inflammatory (markers, HLA-DR, and CD11C) or M2 anti-inflammatory/pro-fibrotic (marker, CD206). Macrophages can switch between the two phenotypes depending on the functional need at the time, M1 and then M2, when tissue repair is required [34]. Furthermore, it is well known that the generation of ROS within macrophages is fundamental to their phagocytic activity, promoting autophagy and the elimination of microbes, debris, and dysfunctional, damaged, and diseased tissue [34,35]. However, it has recently emerged that ROS within macrophages can influence the polarization of different phenotypes, metabolic processes, and disease states [36]. Nitrogen oxides (NOX)-dependent ROS polarized cells to the M2 phenotype and contributed to fatty liver disease [32,36]. It also regulated angiogenesis in disease models of macular degeneration [37]. NOX may also be linked to the NLRP3 inflammasome function, which is essential for triggering inflammatory defence systems in macrophages [38]. The microenvironment of macrophages is a critical determinant in their effector response. The M2 phenotype instructs cells for functional angiogenesis in the normal processes of vessel maturation, healing, growth, and development but non-functional angiogenesis in conditions that promote neoplastic growth [34]. Indeed, the latest evidence also suggests that programming tumor-associated macrophages (TAMs) via ROS can shift to the M1 phenotype, which is associated with a more remarkable ability to block proliferating cancer cells [37]. Levels of ROS can also impact cancer outcome; depending on the stage and treatment, ROS levels either promoted or inhibited cancer cell autophagy [38].

Therefore, we investigated myrrh ethanolic extract by first identifying the key metabolites and then performing in vitro tests to determine its cytotoxic potential in leukemia, breast, and colorectal cancer lines. In addition, the in vitro and in vivo anti-inflammatory activities of myrrh extracts were determined by assessing its effect in promoting phenotypic changes in macrophages, and its effect in mice on wound healing. Finally, the in silico technique was used to predict the safety of the bioactive constituents. Our data will significantly add to the existing body of literature, expanding the scope of research and identifying the therapeutic uses of myrrh while promoting its metabolites for drug discovery.

## 2. Results and Discussion

### 2.1. Metabolite Profiling Using HPLC and QTOF-LCMS

**Figure 1:**
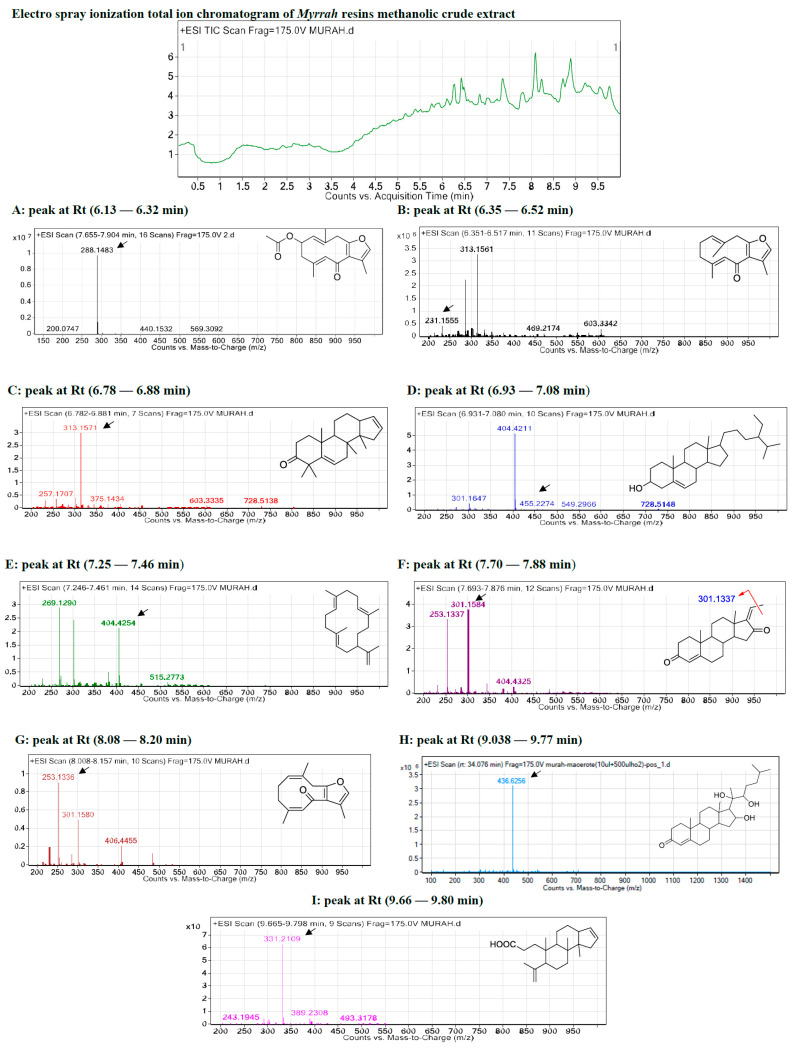
Base peak chromatogram of myrrh resin methanolic extract and identification of secondary metabolites, which are: 4,5-dihydrofuranodienone (**A**), furanodienone (**B**), mansumbinone (**C**), cembrene-A (**D**), campestan-3b-ol (**E**), pregnadiene (**F**), Z,4Z-furanodien-6-one (**G**), guggulsterol (**H**), 3,4-seco-mansumbinoic acid (**I**). Meas *m*/*z*, measured *m*/*z*.

The LC-MS data with molecular features extraction (MFE) algorithm and recursive analysis workflow was used to identify the chemical features of the compounds by screening the detected nodes at various retention times per minute, with a minimum intensity of 6000 counts, which were aligned with previously detected compounds considering the adducts ([M+H]^+^, [M+2H]^+^, [M+Na]^+^ and [M+4H]^+^). The compounds were identified as five steroids: 1. mansumbinone, 2. campestan-3b-ol, 3. pregnadienes, 4. guggulsterols, and 5. 3,4-seco-mansumbinoic acid [29,30]; two diterpenes: camphorene hydrocarbons: 6. Z,4Z-furanodien-6-one and 7. cembrene-A [31,32,33,34]; and two furano-germacranes or furano-bicyclic sesquiterpenoids: 8. 4,5-dihydrofuranodiene and 9. furanodienone. (See Figure 1 and Table 1.)

HPLC/DAD revealed that the myrrh resin methanolic extract was rich in polar and non-polar compounds, with various milli absorbance values (mAbs) ranging from 3000 to 3500 at the wavelengths of 275, 325, and 375 nanometers (nm). Moreover, a group of moderately polar compounds appeared within the retention time of 5.5–6.8 min; this cluster requires further fractionation and orthogonal separation, as this was beyond this project’s scope.

#### 2.1.1. Metabolite A: 4,5-Dihydrofuranodiene

The *m*/*z* values detected for the first peak at retention time 6.13–6.32 min were correlated with the parent compound 4,5-dihydrofuranodiene with *m*/*z* 288.148 and a molecular formula of [C_17_H_20_O_4_] [33], in positive ion mode and [M-H]- with *m*/*z* 287 in negative mode. A high-energy scan produced four fragment ions, [M-CH_3_]+ at *m*/*z* 275.113, [M-2CH_3_]+ at *m*/*z* 258.089, [M-3CH_3_]+ at *m*/*z* 245.118, and [M-CO_2_CH_3_]+ at *m*/*z* 243.118.

4,5-Dihydrofuranodiene is a furano-sesquiterpene with the characteristic 15-carbon germacrane ring structure backbone, built from three isoprene units. Furano-sesquiterpenes from coral were reported to induce apoptosis via a caspase-dependent pathway in leukemia cell lines [34].

#### 2.1.2. Metabolite B: Furanodienone

The *m*/*z* values for the second peak detected at retention time 6.35–6.52 min were correlated with the parent compound furanodienone with *m*/*z* 231.155 and a molecular formula of [C_15_H_18_O_2_] [35,36] in positive ion mode, and [M-H]^−^ with *m*/*z* 229 in negative mode. A high-energy scan, generated three fragment ions [M-CH_3_]^+^ at *m*/*z* 215.107, [M-2CH_3_]+ at *m*/*z* 200.084, and [M-3CH_3_]+ at *m*/*z* 185.023.

Furanodienone is known to have anti-inflammatory, anti-microbial, and anti-cancer properties, commonly found in various species of Curcuma and Commiphora [37,38]. It displayed cytotoxic properties in HER2+ human breast cancer cells by preventing activation of epidermal growth factor receptor (EGFR/HER2) signaling pathways [39,40] and in Michigan Cancer Foundation-7 (MCF7) cells suppressed estrogen receptor alpha (ERα) signaling promoting apoptosis [40]. Furthermore, furanodienone initiated apoptosis through G0/G1 cell cycle arrest in colorectal cancer cell lines via reactive oxygen species/mitogen-activated protein kinases and (ROS/MAPK) caspase-dependent signaling [41].

#### 2.1.3. Metabolite C: Mansumbinone

The *m*/*z* values for the third peak detected at retention time 6.78–6.88 min were correlated with the parent compound mansumbinone with *m*/*z* 313 and a molecular formula of [C_22_H_32_O_2_] [42] in positive ion mode, and [M-H]^−^ with *m*/*z* 311 in negative mode. A high-energy scan generated five fragment ions [M-CH_3_]^+^ at *m*/*z* 297.222, [M-2CH_3_]^+^ at *m*/*z* 282.198, [M-3CH_3_]^+^ at *m*/*z* 267.175, [M-4CH_3_]^+^ at *m*/*z* 252.151, and [M-5CH_3_]^+^ at *m*/*z* 237.128.

Mansumbinone is an octanordammarane triterpene and is known to have anti-inflammatory effects by reducing joint swelling in adjuvant arthritis rats [43,44].

#### 2.1.4. Metabolite D: Cembrene-A

The *m*/*z* values for the fifth peak detected at retention time (7.25–7.46 min) were correlated with the parent compound cembrene-A with *m*/*z* 269.129 and a molecular formula of [C_20_H_30_], in positive ion mode and [M-H]^−^ and with *m*/*z* 267 in negative mode. At a high energy scan five fragment ions were generated [M-CH_3_]^+^ at *m*/*z* 258.235, [M-2CH_3_]^+^ at *m*/*z* 243.211, [M-3CH_3_]^+^ at *m*/*z* 228.188, [M-4CH_3_]^+^ at *m*/*z* 213.164, and [M-C_3_H_5_]^+^ at *m*/*z* 231.211, and *m*/*z* 41.039.

Cembrene-A also known as neocembrene, is an isoprenoid diterpene known to have antioxidant and cytotoxic activities and is also found in soft coral [45].

#### 2.1.5. Metabolite E: Campes-tan-3b-ol

The *m*/*z* values for the fourth peak detected at retention time 6.93–7.08 min were correlated with the parent compound campes-tan-3b-ol with *m*/*z* 404.417 and a molecular formula of [C_28_H_48_O] [42] in positive ion mode and [M-H]- with *m*/*z* 403 in negative mode. In a high-energy scan, six fragment ions were generated: [M-CH_3_]^+^ at *m*/*z* 385.347, [M-2CH_3_]^+^ at *m*/*z* 370.324, [M-3CH_3_]^+^ at *m*/*z* 355.300, [M-4CH_3_]^+^ at *m*/*z* 340.277, [M-_5_CH_3_]^+^ at *m*/*z* 325.253, and [M-C_9_H_19_]^+^ at *m*/*z* 127.149, and *m*/*z* 273.222.

Campes-tan-3b-ol is a phytosterol or ergosterol. Phytosterols, generally known to be a healthy source of fats, and their use is associated with a reduced risk of cancer [46].

#### 2.1.6. Metabolite F: Pregnadienes

The *m*/*z* values for the sixth peak detected at retention time 7.70–7.88 min were correlated with the compound pregnadienes with *m*/*z* 316.240 in positive ion mode and a molecular formula of [C_21_H_32_O_2_] [42] that correlated with a fragmented product of *m*/*z* 301 [C_20_H_27_O_2_]^+^, and [M-H]^−^ with *m*/*z* 311 in negative mode and a high energy scan produced five fragment ions [M-CH_3_]^+^ at *m*/*z* 301.217, [M-2CH_3_]^+^ at *m*/*z* 286.193, and [M-3CH_3_]^+^ at *m*/*z* 271.170.

Pregnadienes are steroids, and their derivatives can mediate anti-inflammatory activity [47]. Furthermore, they may act as 5-alpha-reductase inhibitors [48] and have anti-androgenic effects, which may be useful in cases of hormone-dependent prostate cancer [49].

#### 2.1.7. Metabolite G: Z,4Z-Furanodien-6-one

The *m*/*z* values for the seventh peak detected at retention time 8.08–8.20 min were correlated with the parent compound Z,4Z-furanodien-6-one [50,51] with sodiated *m*/*z* 253.124 and a molecular formula of [C_15_H_18_O_2_], in positive ion mode and [M-H]^−^ with *m*/*z* 243 in negative mode; the high-energy scan generated three fragment ions [M-CH_3_]^+^ at *m*/*z* 238.131, [M-2CH_3_]^+^ at *m*/*z* 223.107, and [M-3CH_3_]^+^ at *m*/*z* 208.084.

Z,4Z-Furanodien-6-one is a type of furano-sesquiterpene known as furanogermacrene. It can be found in many plants, including ginger, which may contributes to strong anti-inflammatory effects via inhibition of Prostaglandin E2 (PGE2) [52]. In addition, it has been shown to have in vitro anti-plasmodial effects [53] and was found to have neuroprotective properties against matrix metalloproteinase (MMP)-induced neuronal cell death [54].

#### 2.1.8. Metabolite H: Guggulsterone

The *m*/*z* values for the eighth peak detected at retention time 4.68–4.77 min were correlated with the parent compound guggulsterols [55] with *m*/*z* [M+Na]^+^ 455, and 432.329 and a molecular formula of [C_27_H_44_O_4_], in positive ion mode and [M-H]^−^ with *m*/*z* 431 in negative mode. A high energy scan generated six fragment ions [M-CH_3_]^+^ at *m*/*z* 402.266, [M-2CH_3_]^+^ at *m*/*z* 388.243, [M-3CH_3_]^+^ at *m*/*z* 355.300, [M-4CH_3_]^+^ at *m*/*z* 344.243, [M-5CH_3_]^+^ at *m*/*z* 329.219, and [M-C8H17]^+^ at *m*/*z* 273.222, and *m*/*z* 113.133.

Guggulsterone is a phytosterol also found in the resin of the guggul plant. It has various pharmacological properties, from cholesterol and bile metabolism to its role in modulating protein expression of pro-inflammatory proteins such as nuclear factor kappa-light-chain-enhancer of activated B cells, (NFKB) and events necessary in tumorigenesis and chemoresistant tumors [56].

#### 2.1.9. Metabolite I: 3,4-Seco-mansumbinoic Acid

The *m*/*z* values for the ninth peak detected at a retention time (9.66–9.80 min) were correlated with the parent compound 3,4-seco-mansumbinoic acid [55] with *m*/*z* 331.2097 and a molecular formula of [C_22_H_34_O_2_], in positive ion mode and [M-H]^−^ with *m*/*z* 329 in negative mode and a high-energy scan produced six fragment ions [M-CH_3_]^+^ at *m*/*z* 315.232, [M-2CH_3_]^+^ at *m*/*z* 300.209, [M-3CH_3_]^+^ at *m*/*z* 285.185, and [M-C_2_H_3_O_2_]^+^ at *m*/*z* 271.243. 

3,4-Seco-mansumbinoic acid is an octanordammarane with anti-bacterial properties, and is reported to be more potent than norfloxacin [57].

### 2.2. Anti-Cancer Activity of Myrrh Resin Extract

**Figure 2:**
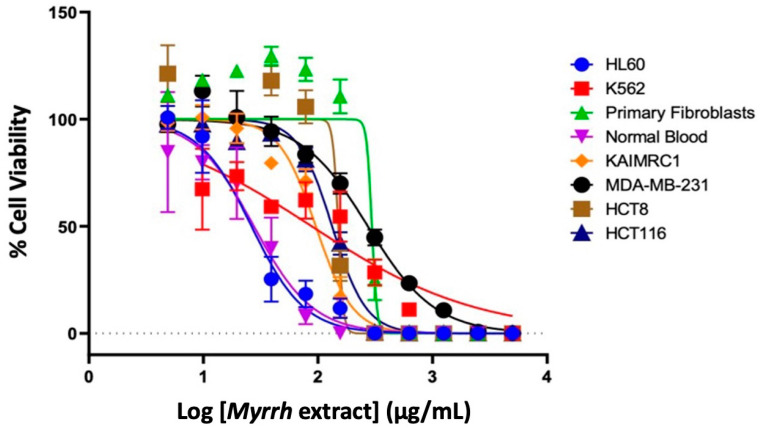
Treatment of leukemia, breast, and colorectal cancer cell lines with myrrh resin extract. Cell viability was assessed by TiterGlo™ assay, and serial dilution was used to calculate the IC_50_ values. The *X*-axis presents the log of myrrh concentration in µg/mL, and the *Y*-axis presents normalized absorbance as a percentage.

The U.S. National Cancer Institute (NCI) has established criteria of in vitro cytotoxicity for crude extracts in preliminary assays, which is an IC_50_ value of less than 30 μg/mL. In this experiment, we sought to determine the cytotoxic activity of myrrh resin extract in breast cancer cell lines (MDA-MB-231 and KAIMRC1), colorectal cancer cells (HCT8 and HCT116), leukemia cell lines (HL60 and K562), and control cells: normal primary fibroblasts and normal blood (P1 and N1) [58]. Cells were treated with various concentrations of myrrh extract to generate a functional dose–response curve, from which the IC_50_ (µg/mL) value for each cell line was calculated (Figure 2). We categorized the IC_50_ values as potent cytotoxic effect (IC_50_ < 10 μg/mL, moderate cytotoxic effect (IC_50_: 11–100 μg/mL), and poor cytotoxic effect (IC_50_ > 100 μg/mL). 

**Figure 3:**
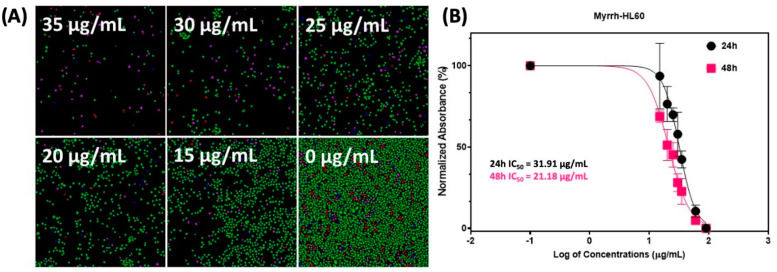
Treatment of leukemic HL60 cell line with myrrh extract. (**A**) High content imaging of treated cells at 48 h. The cell health module was used to identify live (green), pre-apoptotic (blue), post-apoptotic (pink), and dead (red) cells. (**B**) Cell viability was assessed by TiterGlo™ assay at 24 h and 48 h. Five points, i.e., 35, 30, 25, 20, and 15 µg/mL serial dilution, were used to calculate the IC_50_ values (shown in the figure). The *X*-axis presents the log of myrrh concentration in µg/mL, and the *Y*-axis represents the percentage cell viability.

The results in Table 2 show that the myrrh extract had moderate anti-cancer activity against both the leukemia cell lines, HL60 (26.29 μg/mL) and K562 (88.27 μg/mL), and one breast cancer cell line, KAIMRC1 (95.73 μg/mL). In comparison, IC_50_ values were above 100 μg/mL for both colorectal cancer cell lines and normal primary fibroblasts and thus were poorly cytotoxic. Myrrh extract was also moderately cytotoxic to normal blood cells (27.59 μg/mL) [59]. The results of the TiterGlo cytotoxicity assay were further confirmed using high content imaging that showed that myrrh treatment caused a dose-dependent cytotoxicity in HL60 leukemia cells (Figure 3).

There are many studies that have tested fractions of the myrrh extract or isolated and identified components within the extract and tested their cytotoxic potential on various cancer cells. Gao et al., 2020 reported significant cytotoxic effects of myrrh ethanol extracts (MYC) on human multiple myeloma cells (U266) [60,61,62,63,64]. Su et al., 2011 showed that myrrh extracts significantly inhibited the cell proliferation of three gynecological cell lines (A2780, SK-VO-3, and Shikawa) [65]. Likewise, breast cancer cells (MCF-7) and skin cancer cells (HS-1) were also sensitive to myrrh essential oil [40]. Components found in myrrh, such as the monoterpenoid compound B-elemene and the furano-sesquiterpenoids (CM1) and 2-acetoxyfuranodiene (CM2) are known to inhibit numerous cancer cell lines [8,58,59,60]. CMI and CM2 had a potent cytotoxic effect in human liver carcinoma cells (HepG2) and breast cancer cells (MCF7), with IC_50_ values of 3.6 μM and 4.4 μM, respectively [8]. In addition, triterpenoids isolated from *Myrrh* had significant cytotoxic effects on human prostate cancer (PC3) and (DU145) cells, with IC_50_ values ranging from 9.6 to 37.2 μM [24,61,62]. Studies have also reported the potential anti-tumor activity of myrrh steroid guggulsterone (GS) [64]. In agreement with the published literature, we found that myrrh methanolic extract exhibited a moderate anti-cancer activity in three cancer cell lines: the leukemia cell lines HLC60 and K562, and the breast cancer cell line KAIMRC1. Although we did not test the individual components of myrrh, it is reasonable to assume that many of the isoprenoids and steroids found in myrrh are contribute to its cytotoxic potential. Very few studies have demonstrated the selective therapeutic potential of myrrh by comparable testing in control cells and or performing in vitro toxicity experiments. The current cytotoxicity data describe the myrrh extract as selectively toxic to different types of cancer cells and normal cells. Despite this, it is imperative to test the extracts’ in vitro toxicity to determine their true therapeutic potential. In addition, purification and individual testing of the metabolites’ cytotoxicity are required to uncover the specific roles of myrrh metabolites in cancer therapy. We have led the way in recognizing the true therapeutic potential of the extract by identifying the metabolites and performing in silico predictions of their safety profile, which will pave the way for future in vitro and in vivo experiments [10,66,67,68].

### 2.3. In Vitro Anti-Inflammatory Activity Investigation

**Figure 4:**
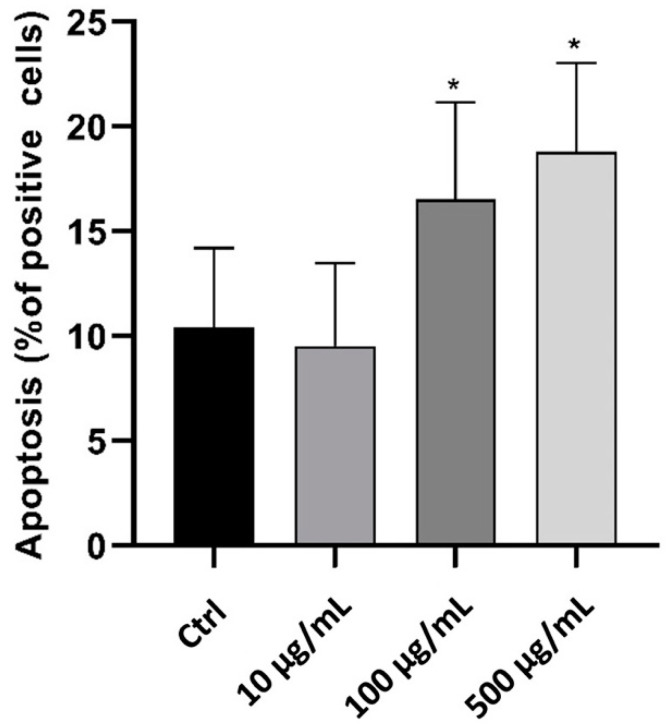
In vitro evaluation of the effect of myrrh methanolic extract on cell apoptosis using Thp-1-like macrophage cells (* *p* < 0.05).

**Figure 5:**
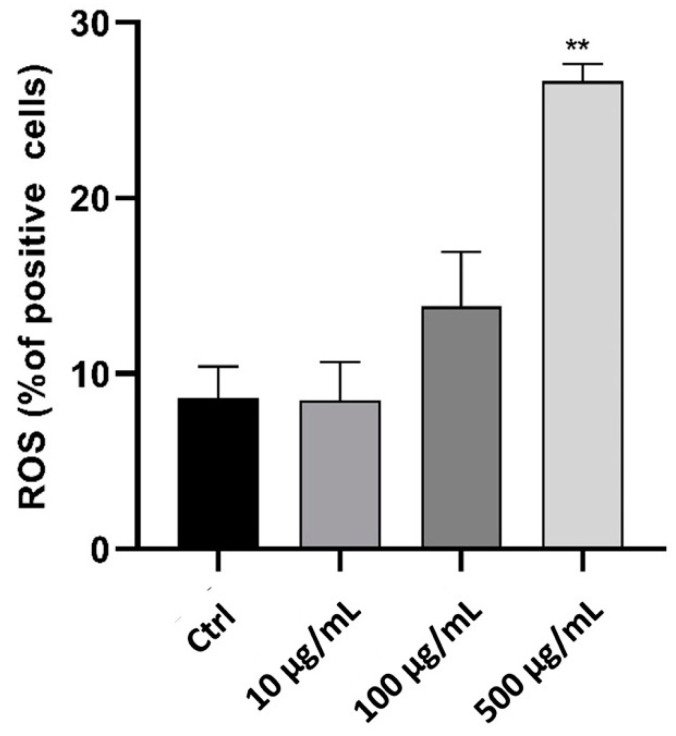
Effect of myrrh methanolic extract on reactive oxygen species production in Thp-1-like macrophage cells. The results demonstrate a dose-dependent increase following 100 μg treatment, with a significant change observed at 500 μg treatment (** *p* < 0.01).

To examine the effect of myrrh extract on inflammatory cells, we used the phagocytic human leukemic monocytic cell line, Tamm-Horsfall Protein 1 (Thp-1)-like macrophage cells, which can differentiate into macrophage-like cells by the activation of protein kinase C upon treatment with PMA. These cells are readily used as a model to study monocyte/macrophage functions and their signalling pathways. We used Annexin V staining to identify the effect of myrrh extract treatment on apoptosis, as shown in Figure 4. Treatment with 100 μg/mL significantly increased the percentage of apoptotic cells (*p* < 0.05). Since oxidative stress plays a crucial role in the pathophysiology of different diseases and modulates cell function and structure, we measured ROS to test whether myrrh treatment impacted the oxidant/antioxidant balance and generated ROS in Thp-1 cells [69,70]. Our results showed a dose-dependent increase in ROS following 100 μg/mL treatment with myrrh, and a significant increase was observed at the highest concentration tested, 500 μg/mL (*p* < 0.01) (Figure 5).

THP-1 cells (ATCC, TIB-202) were cultured in RPMI (10% FBS (Gibco) supplemented with 2-mercaptoethanol to a final concentration of 0.05 mM and 1% pen/strep antibiotic (Gibco) then incubated at 37 °C, 5% CO_2_ in humidified incubator. For cell (of passage 16 or below) differentiation, phorbol-12-myristate 13-acetate (PMA, 10 nM) (Sigma-Aldrich Burlington, MA, United States) was used for 72 h. To remove the excess PMA, the cells were washed three times with phosphate-buffered saline (PBS). After 3 days, the cells become fully differentiated to a macrophage-like phenotype and are ready for experiments. Following the treatment with myrrh, the cells were analyzed using flow cytometry as described previously (34616396). In brief, cells were resuspended in 100 µL FACS buffer (PBS 5% FBS), stained with fluorescent antibodies at 1 µg/mL as PE-conjugated antibodies specific for CD11C, APC-conjugated specific for HLA-DR and PE-conjugated specific for CD206. CD11C and HLA-DR are used to characterize M1-like macrophages, whereas CD206 was used to identify the M2-like phenotype. Cells were incubated in the dark for 1 h at 4 °C, then washed three times to remove the background, and resuspended in 400 µL of FACS buffer [71,72]. Detection of fluorescence was acquired via flow cytometry (BDFACS contoII and LSR Fortessa) (BD Bioscience) and data analysis was performed using Diva software (BD Bioscience) [73].

**Figure 6:**
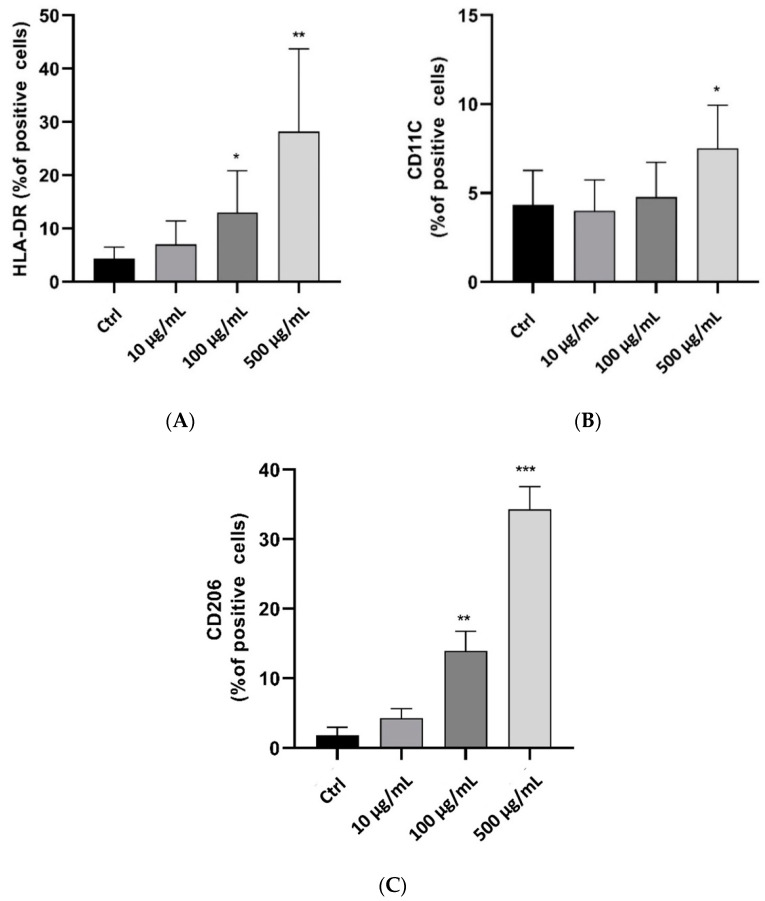
Treatment of Thp-1-like macrophage cells with myrrh extract and the effect on M1 or M2-like phenotypic cell surface markers. In vitro dose-dependent activation of M1 phenotype markers on HLA-DR (**A**) and CD11C (**B**) following treatment with myrrh extract. Dose-dependent induction of CD206 M2-like-phenotype marker following in vitro treatment with myrrh methanolic extract (**C**) (* *p* < 0.05, ** *p* < 0.01, *** *p* < 0.005).

In addition, phenotypic changes and plasticity are significant characteristics of Thp-1-like macrophage cells. Differences in exogenous stimulators secreted by other cells or components of the microenvironment can influence their final phenotype. Our findings showed that treatment with myrrh increased both M1-like-phenotype markers, human leukocyte antigen–DR isotype (HLA-DR) (27%) and CD11C (12%) (Figure 6A,B), and the M2-like-phenotype marker CD206 (33%) (Figure 6C); however, the percentage of M2-like cells was greater with treatments of 100 μg/mL and 500 μg/mL, indicating that the anti-inflammatory response was greater than the pro-inflammatory response with myrrh treatment. The increase in apoptosis may be related to the final effect of clearance, as is typically seen in vitro after polarization and response to inflammatory or infectious stimuli [74].

Together, our data suggest a significant effect of myrrh to promote both M1 and M2 phenotypes and generate ROS. Thus, myrrh appears to modulate macrophage function. The limitation of our data is that single stains were performed so the ROS generation described here is for the mixed population and cannot be ascribed to any specific subtype. It is well known that ROS production is typical of the M1 phenotype involved in pro-inflammatory responses and protection against bacterial and viral infections and is regarded as anti-neoplastic [75], whereas the M2 type is essential for healing, pro-fibrotic, and anti-inflammatory effects. In the case of breast cancer, ROS has been detected in both M1 and M2 types of macrophages, but to a lesser degree in M2 where it appears to be essential for its polarisation to the TAM-like M2 phenotype. Furthermore, reducing ROS inhibits characteristic M2-like gene expression [76]. Similarly, in colon cancer, the chemotherapeutic agent fenretinide inhibited M2-like macrophages [77]. Indeed, the modulation of macrophage polarization is an important chemotherapeutic strategy [78,79,80,81]. However, it is important to note the differences in subsets of the M2 macrophages that are described to be of various effector classes based on the phenotypic expression markers. In vitro, we detected both pro-inflammatory, anti-neoplastic (M1) and anti-inflammatory, pro-neoplastic (M2) cells, with a slightly higher proportion of the letter. Due to limitations in the generated data, we can only conclude that there is potential for myrrh to be both anti-neoplastic and anti-inflammatory, and it may be that the microenvironment is a key determinant of its typical effect. Further in-depth studies are required, and in vivo studies assessing macrophage modulation in normal and disease models are a likely starting point to shed light on the precise effects of myrrh. Another point to consider is that there is much information describing myrrh as anti-inflammatory and immunomodulatory, influencing the activity of many different types of immune cells, including activated macrophages [82], where myrrh attenuated the expression of pro-inflammatory mediators CXCL13 and TNFα [83,84,85,86]. Similarly, there are natural phytochemicals including triterpenoids, sterols, anthraquinones, flavonoids, and numerous other bioactive compounds derived from herbs and plants that modulate M1 and M2 macrophage polarization and function [87]. Thus, myrrh could be a promising herb in treating cancer, pro-inflammatory conditions, and infections [88].

### 2.4. In Vivo Anti-Inflammatory and Wound-Healing Activity Investigation

**Figure 7:**
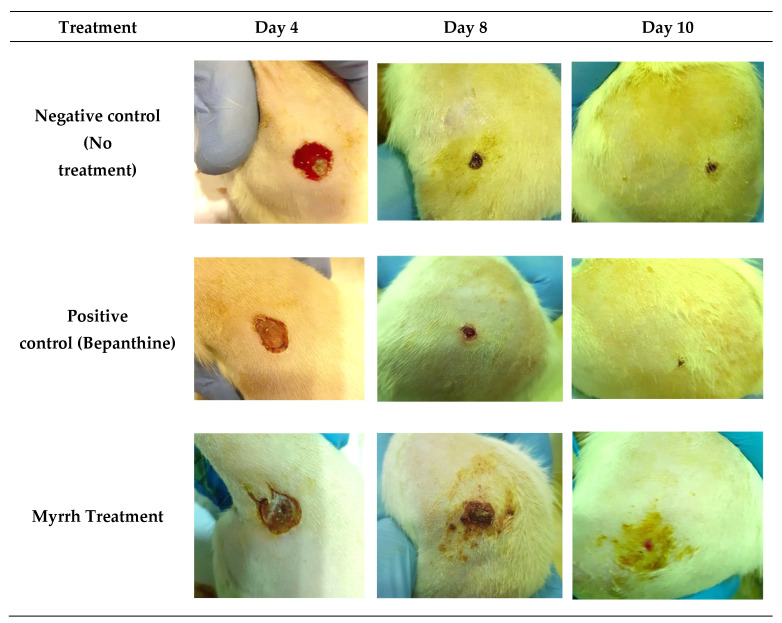
In vivo testing of the anti-inflammatory and wound-healing activity of negative control, bepanthine, and myrrh treatment. Myrrh and bepanthine were noted to enhance wound contraction, hair growth, and proliferative stage maturation of the blood vessels.

The anti-inflammatory and wound-healing activity of myrrh extract was investigated. Rats were divided into three groups—negative control (no treatment), positive control (bepanthine), and treatment group (myrrh)—and were wounded as described in the materials and methods. After inducing the wound, rats were assessed on days 4, 8, and 10. As shown in Figure 7, rats in all three groups were at the same stage of wound healing, which is hemostasis, in which the bleeding had stopped, a blood clot had developed, and fibrin had formed. Our findings revealed that bepanthine treatment showed the highest contraction compared to the myrrh treatment group and control group. This might be explained by the presence of dexapanthol, which helps to oxygenate the skin and is vital for metabolic processes. The application of myrrh treatment caused a wound contraction in addition to promoting hair growth and proliferative stage maturation of the blood vessels, which was also observed with bepanthine treatment compared to the negative control.

**Figure 8:**
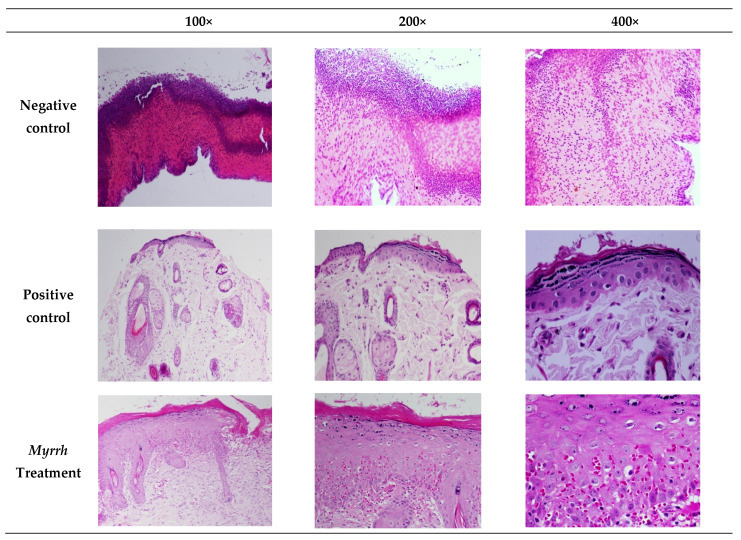
The histological alteration of rat wounds treated with myrrh methanolic extract, bepanthine, and the negative control. Bepanthine- and myrrh-treated wound revealed normal epidermal cells and re-epithelialization comparable to the negative control group.

Histological alterations occur throughout the wound-healing process; thus we examined whether myrrh promoted a significant benefit in wound healing at the cellular level, as shown in Figure 8. In the wound treated with myrrh, the proliferation of fibroblast cells, epithelial degeneration, and normal epidermal cells were evident. Moreover, re-epithelialization and epidermal hyper-thickening in the wound area had occurred. In contrast, bepanthine demonstrated normal skin tissues with the removal of mild keratinized tissue. Furthermore, a dense layer of the epidermis grew with granular tissue that revealed cells and arteries filling the wound. On the contrary, the negative control revealed distributed inflammatory cells and bleeding, especially neutrophils within the loose connective tissue. The existence of immature granulation tissue indicates tissue injury with severe inflammatory cell diffusion, and the presence of inflammatory cells in the scar tissue was noted. During the normal process of wound healing, there is a phenotypic transition of the macrophages from the M1 pro-inflammatory to the M2 anti-inflammatory and pro-fibrotic type [82]. As described earlier, M2 cells promote the formation of the extracellular matrix and secrete PDGF required for angiogenesis and normal tissue function [83], and the depletion of macrophages is associated with delayed re-epithelialization, impaired angiogenesis, and diminished collagen formation [84]. Therefore, the effect of myrrh treatment is likely to be enhancing normal M2 function. Previous reports describe myrrh along with other herbal extracts to promote wound contraction fibrosis and wound healing in rats [85].

### 2.5. In Silico Computational Investigation

#### 2.5.1. Activity Prediction

To investigate the anti-inflammatory and anti-cancer properties of myrrh methanolic extract, nine bioactive compounds were examined using the PASS online webserver to predict their antioxidant, anti-inflammatory, wound-healing, and anti-neoplastic activities. The higher P_a_ and lower P_i_ values indicate a higher probability that this compound possesses biological activity when tested experimentally. As demonstrated in Table 3, compound 4 has the highest anti-inflammatory score with P_a_ of 0.602. In addition, compound 1 demonstrated the highest antioxidant predicted activity with P_a_ of 0.324. Moreover, compound 5 had the highest probability of being active as a wound-healing agent with a P_a_ score of 0.416. Additionally, compounds 1, 2, and 7 were the only agents that exhibited free radical scavenging potential. Regarding the anti-neoplastic properties of myrrh’s compounds, compound 6 had the highest predicted activity with a P_a_ score of 0.888. The high P_a_ values for some metabolites suggest that these compounds could potentially mediate anti-inflammatory, wound-healing effects, free radical scavenging activity, and anti-neoplastic properties of myrrh extracts.

#### 2.5.2. Target Prediction

##### Molinspiration

The Molinspiration web server predicts and identifies compounds’ specific biological activity against targets. A bioactivity score greater than 0.00 is more likely to show substantial biological activity. In contrast, scores of −0.50 to 0.00 indicate moderate activity, and a score below −0.50 is categorized as inactive [79]. The bioactivity score was measured for the six main targets described below and summarized in Table 4.

##### GPCR Ligand

G-protein-coupled receptors are an effective drug target for around 50 percent of marketed drugs and treatments owing to their participation in signaling pathways linked to various types of cancer [80]. As illustrated in Table 4, compounds 3, 4, 5, 8, and 9 have good activity as G-protein-coupled receptors.

##### Ion Channel Modulator

Ion channels are a vast category of important proteins involved in an array of normal physiological processes. They are also reported to be involved in tumor vascularization, modulation, and proliferation, and may be a potential target for tumor cells [81]. Compounds 4, 5, 8, and 9 showed moderate activity targeting the ion channel modulator.

##### Kinase Inhibitor

Human cancer initiation and development are strongly correlated with the action or dysfunction of kinases. The small-molecule kinase inhibitors showed great promise and potential in clinical therapy for the treatment of different types of cancer [82]. Remarkably, protein kinases are considered the second most targeted class of drug targets [83]. All compounds were inactive at this target.

##### Nuclear Receptor Ligand

Nuclear receptors are dominant regulators of physiological processes and are known to display pro-oncogenic activities in tumor cells [84]. All compounds appear to have a significant role as nuclear receptor targets, suggesting that they may contribute to the anti-cancer activity of myrrh resin, except for compound 2 and 7. Furthermore, a similar finding has been demonstrated by Wang Xi et al., 2011, who found that sesquiterpenoids from myrrh inhibited androgen receptors by possibly regulating nuclear androgen receptor translocation or interrupting the interaction between androgen receptors and its coactivators androgen receptor-associated protein 70 (ARA70) and steroid receptor coactivator-1 (SRC-1). Sesquiterpenoids from myrrh could be implemented as novel chemotherapeutic drugs for prostate cancer treatment [85].

##### Protease Inhibitor

Proteases are also involved in the growth and development of tumors that are highly dependent on the supply of nutrients and oxygen. Therefore, protease inhibitors may be used as a powerful cancer therapy strategy [37,86]. Compounds 5 and 8 were the only active compounds with good protease inhibitor targeting.

##### Enzyme Inhibitor

Enzymes help to accelerate chemical reactions in the organs, including cell signaling, growth, and separation and engage in several cell functions. Enzyme inhibitors can be used in chemotherapeutic agents to suppress enzymes required for cancer cell growth [11]. Interestingly, all the compounds showed high scores and good activity as enzyme inhibitors, indicating that enzymes may also be important in mediating myrrh extract cytotoxicity. Interestingly, a recent study reported that myrrh could suppress stomach cancer cell proliferation and metastasis and induce cell death by inhibiting the upregulation of the enzyme cyclooxygenase 2 (COX-2) [46].

##### SEA Search and Swiss Target Predictions

The SEA Search Server predicts the possibility of specific proteins being targets for the compounds. A high similarity score (maxTC) with a low significance score (*p*-value) indicates a greater chance that the protein is a potential target. As shown in Table 5, compound 6 has the highest probability (maxTC values of 1.00) that it may target the glucocorticoid and androgen receptors. Moreover, compound 5 was predicted to only target the androgen receptor, with a maxTC value of 0.53. Additionally, compound 8 was predicted to target both glucocorticoid and androgen receptors, with maxTC values of 0.40 and 0.53, respectively.

Furthermore, Swiss Target prediction identified compounds 2, 7, 8, and 9 as able to target cyclooxygenase-2 with probability values between 0.119 to 0.049, with the highest value attributed to compound 8. Moreover, compounds 3, 6, 8, and 9 were predicted to target the glucocorticoids receptor with a probability between 1.0 to 0.102. Additionally, compounds 2 and 7 were predicted to target beta-amyloid A4 protein with a probability of 0.049 for both compounds. All compounds were predicted to target the androgen receptors, except compounds 2, 4, and 7, with excellent values between 1.0 to 0.098. Finally, only compound 9 was expected to target topoisomerase I with a probability value of 0.105.

#### 2.5.3. ADME Prediction

A compound’s molecular weight (MW) is associated with and predicts properties such as passive diffusion, hydrophobicity, and cellular membrane permeability. For instance, compared to extremely high molecular weight, low MW correlated with high gastrointestinal (GI) absorption and might show some water solubility [86]. This suggests that regulating physicochemical properties, such as lipid solubility, can strengthen compound safety and the effectiveness of therapeutic success within a specified optimum range.

One study attributed the cytotoxic activity of myrrh on four human gynecological cancer cell lines to the fat-soluble extracts and chemical components of myrrh [87]. In describing the chemical components of myrrh, the degree of solubility of the components was the primary requirement for achieving optimal concentration in the bloodstream to allow a pharmacological therapeutic effect to be observed [88]. Therefore, the agent to be absorbed must persist in solution at the absorption site. The most frequent consequences of low oral bioavailability are low solubility and permeability [45]. Fortunately, hydrogen bond acceptors can enhance the molecules’ solubility, which in turn helps to improve the oral bioavailability of drugs [89].

Based on Lipinski’s rule, our computational results demonstrated that the identified myrrh compounds possess a molecular weight below 500 g/mol, increasing their expected value of being absorbed orally in the gastrointestinal tract [90].

The lipophilic value can be theoretically calculated using its log *p* value (logarithm partition coefficient). The recommended log *p* values when using SwissADME should be within −2.0–6.5 to be accepted as a good candidate for oral absorption [91]. Only compound 5 was not within the referenced range, which was predictive of poor GI absorption. All other compounds fell within the recommended ranges and thus may be adequately absorbed through oral consumption.

Poor water solubility is correlated with low absorption [92]. The primary goal is therefore to avoid poorly soluble compounds [93]. According to SwissADME, all compounds were within the acceptable boundaries of −6.5–0.5; thus, they are all predicted to be soluble.

From the active constituents present in the myrrh resin extract, only compounds 3, 4, 5, and 8 are potentially not permeable to the blood-brain barrier (BBB). Moreover, these results correlated well with Log S and Log P as these compounds exhibited modest water-solubility and good lipophilicity, suggesting the ability to cross the BBB easily.

Hydrogen bond donor and acceptor (HBD/HBA): All myrrh derivatives aligned with Lipinski’s rule based on the number of hydrogen bond donors and acceptors, except compounds 3, 4, and 5, which have less than one hydrogen bond acceptor suggesting that they would be the least basic compounds [94]. All ADME properties are summarized in Table 6.

#### 2.5.4. CYP Inhibition Profile

Herbs can enhance or reduce the predicted activity of prescribed drugs by interacting with CYP450 enzymes causing undesired side effects, toxicity, or therapeutic failure [49]. More than 50 CYP450 enzymes are known, yet CYP1A2, CYP2C9, CYP2C19, CYP2D6, and CYP3A4 are responsible for the metabolism of 90% of drugs [95,96]. As shown in Table 7, only compounds 5 and 8 were not predicted to inhibit CYP enzymes. Moreover, no compounds were predicted to inhibit CYP1A2 except compounds 2, 7, and 9. No compounds are expected to inhibit CYP2C9 except for 3, 4, and 6. Additionally, no compounds were predicted to inhibit CYP2D6 and CYP3A4.

#### 2.5.5. Organ and End-Point Toxicity Prediction

ProTox-II webserver is an in silico model utilized to investigate the toxicity of chemicals based on chemical and molecular target knowledge, including oral toxicity, organ toxicity (hepatotoxicity), and the toxicological endpoints, including mutagenicity, carcinogenicity, and immunotoxicity. Compound 3 was the safest compound, with a predicted lethal dose (LD_50_) value of 15,000 mg/kg. All the remaining compounds were in class 3–5, which are relatively safe from an oral toxicity perspective, except for compound 9, which was class 2. Furthermore, compounds 6 and 8 could be carcinogenic, whereas compounds 5, 6, and 8 are considered immunogenic. Lastly, only compound 9 is predicted to be hepatotoxic (See Table 8) for color code key at (Table 9).

## 3. Materials and Methods

### 3.1. Plant Material and Extraction

Myrrh resins were purchased from a nursery house in Riyadh, Saudi Arabia. The resins were purchased with an official certificate of authentication and purity percentage certificate number RY03-95-19. The resins were ground, soaked in methanol 95% for 24 h, then filtered. The collected supernatant layer was concentrated at 100 mL using a rotary evaporator. The weight of the discarded filtrate was 80 gm. The extract was collected, measured, and dried using anhydrous sodium sulfate and stored at 4 °C for further analysis.

### 3.2. Metabolites Profiling Using HPLC and QTOF-LCMS

The methanolic extract of myrrh was evaporated to dryness, then subjected to analysis by analytical reversed phase-high performance liquid chromatographic–diode array detection (RP-HPLC–DAD) followed by iquid chromatography–mass spectrometry (LC/MS). The extract analysis was performed on the Agilent 1260 Infinity HPLC system (Agilent, Böblingen, Germany) coupled with the Agilent 6530 Quadrupole Time of Flight (Agilent, Singapore). Separation was performed using Agilent Extend-C18 column (2.1 mm × 50 mm, 1.8 μm) with the following elution gradient; 0–1 min, 5% B; 1–11 min, 5–100% B; 11–13 min, 95%B; 13–15 min, 5%B; 15–16 min, 5%B using mobile phase A (0.1% HCOOH in water) and mobile phase B (0.1% HCOOH in methanol). The injection volume was 10 µL and the flow rate was 300 µL/min. The acquisition method MS1 was achieved in positive mode in the mass range from 100–600 *m*/*z*. The mass spectrometer parameters were set as follows: gas temperature = 300 °C; gas flow = 8 I/min; nebulizer = 35 psig; sheath gas temperature = 350, and sheath gas flow rate = 11. The MS/MS fragmentation of the identified nine compounds was conducted using a similar protocol applying soft fragmentation energy (20 eV).

### 3.3. In Vitro Anticancer Activity of Myrrh Resins Extract

#### 3.3.1. Cell Viability Assay

Cytotoxicity assays were used to evaluate the effectiveness of drug candidates on cancer cells in vitro. The breast cancer, normal primary fibroblast, and colorectal cancer cell lines used in the study were purchased from ATCC, USA. KAIMRC1 was isolated and established in the core laboratory facility of KAIMRC, Riyadh, Saudi Arabia. DMEM containing 10% FBS, 1% L-glutamine, and 1% antibiotics (Pen-Strep) was used to maintain the breast cancer, normal primary fibroblast, and colorectal cancer cell lines, whereas leukemia and normal blood cells were maintained in advanced RPMI containing 10% FBS, 1% 406 L-glutamine, and 1% antibiotics (Pen-Strep). On the first day, cells were plated on flat-bottomed white 96-well plates at 5 × 10^3^ cells/well density in 100 μL growth medium. The following day, serial dilutions of myrrh extract, ranging from 5 mg to 0.01 μg/100 μL cell culture medium, were made in triplicate and were added to the cells and incubated at 37 °C with 5% CO_2_ for 48 h. Cell viability was determined using the CellTiter-Glo assay (Promega) following the manufacturer’s protocol. The luminescence was measured on the Envision plate reader (Perkin Elmer). Luminescence readings were normalized to averaged dimethyl sulfoxide (DMSO) controls and expressed as a relative percentage.

#### 3.3.2. High Content Imaging (HCI) Assay

HL-60 cells were plated in 96-well plates at a density of 10,000 cells per well. Cells were treated with five graded concentrations of myrrh, 35, 30, 25, 20, and 15 μg/mL serial dilution. After treatment, cells were stained with HOECHST33342 (2.5 μg/mL), Yo-Pro-I (2.5 μg/mL), and propidium iodide (2.5 μg/mL) for 10 min at 37 °C and 5% CO_2_. Plates were imaged using a Molecular Devices ImageXpress^®^ Microsystem and analyzed using MetaXpress^®^ software, Molecular Devices, Downingtown, PA, USA. The Cell Health module available in the MetaXpress software was used to count live, apoptotic and dead cells.

### 3.4. In Vitro Anti-Inflammatory Activity Investigation

#### 3.4.1. Cell Lines

Macrophages were differentiated from THP-1 cells (ATCC, TIB-202) following phorbol-12-myristate 13-acetate (PMA, 10 nM) (Sigma-Aldrich, Burlington, MA, United States) stimulation for 3 days. After washing, cells were cultured in RPMI 1640 with 2-mercaptoethanol 0.05 mM, 1% penicillin/streptomycin antibiotic (Gibco), and 10% fetal bovine serum (Gibco) and incubated in a humidified 37 °C, 5% CO_2_ incubator. After 24 h of resting, cells were treated with *Myrrh* Resin Methanolic Extract (10, 100, and 500 μg/mL) for another 48 h.

#### 3.4.2. Apoptosis Assay

After treatment, to evaluate apoptosis, cells were washed twice with cold phosphate buffered saline (PBS) before resuspension in 1×Annexin-V binding buffer (Thermo Fisher) at a concentration of 1 × 10^6^ cells/mL. Cells were diluted by 100 × 10^3^, and purified recombinant Annexin V-FITC (10 μg) was used to detect the phosphatidylserine during apoptosis.

#### 3.4.3. Measurement of ROS Levels

CellROX™ (ThermoFisher scientific, Burlington, MA, United States) Deep Red fluorogenic probe was used to detect reactive oxygen species (ROS) production. Briefly, cells were incubated for 25 min with CellROX (4μM) at 37 °C in the dark. ROS production was measured using a BD FACSCanto II flow cytometer at 644/665 nm of excitation/emission wavelength.

### 3.5. In Vivo Anti-Inflammatory Activity Investigation

The present investigation involved male Wistar rats (8 weeks old: 220–250 g), provided by Experimental Animal Care Center, Pharmacy College, King Saud University. Rats were separated into three groups of nine animals each. An inhaled anesthetic (Diethyl-Ether) was utilized to anesthetize the animals. Before wounding, they were depilated at the predestined area, and a wound was made by removing a 1 cm full thickness of the allocated site on the lower back above the tail. The treatment of rats involved the topical application of the negative control group (no treatment), the positive control group (bepanthine ointment), and the myrrh treatment group was applied as 2 mG/0.5 mL. All treatments were applied to corresponding groups once every other day for 13 days, starting from the day of injury (Day 0).

### 3.6. In Silico Computational Investigation

#### 3.6.1. Activity Prediction

The active compounds were prepared by generating the simplified molecular-input line-entry system (SMILES) from the 2 dimensional (2D) chemical structure, followed by computational prediction of their anti-oxidant, anti-inflammatory, and wound-healing activities using the online service Prediction of Activity Spectra for Substances (PASS) [84]. This web server provides predictions for biological activities using models obtained from bioactive ligands to find the probability of a molecule being active (P_a_) or inactive (P_i_). Furthermore, the scores of P_a_ and P_i_ ranged from 0 to 1, in which a compound with a score of P_a_ > P_i_ can be considered active [97]. Moreover, P_a_ > 0.7 demonstrates a high activity, P_a_ ranging from 0.7 to 0.5 reflects a moderate activity, and a score of P_a_ < 0.5 indicates a low chance of activity.

#### 3.6.2. Target Prediction

##### Molinspiration

The prepared SMILES were also used in the Molinspration webserver to calculate the bioactivity scores for each active compound with various molecular targets. Moreover, the bioactivity scores represent predicted activity at a specific target. The larger the bioavailability score, the higher the compound’s probability of being active at a specified target [98].

##### SEA Search

Herein, we investigated the target prediction of *Myrrh’s* compounds activity using the similarity ensemble approach (SEA) search server, which conducts a quantitative classification and target association according to the chemical similarity of protein-related ligands and also creates a list of Max Tanimoto coefficients (MaxTc) and E-values [99].

##### Swiss Target Prediction

To further investigate the targets for myrrh’s compounds, we performed a target prediction using Swiss Target that precisely predicts the targets of bioactive compounds based on 2D and 3D similarity measures with known ligands [61].

#### 3.6.3. ADME Prediction

The calculations of the absorption, distribution, metabolism, and elimination (ADME) properties of the myrrh active derivatives were performed using the SwissADME web server. The SMILES of each active compound were used as inputs to generate ADME data, and the results were then compared and analyzed according to the reported literature values [63].

#### 3.6.4. CYP Inhibition Profile

The SwissADME server predicted the cytochrome P450 enzyme inhibition profile for each myrrh bioactive compound. Several cytochromes P450 (CYP) are involved in interactions with xenobiotics such as CYP1A2, CYP2C19, CYP2C9, CYP2D6, and CYP3A4 [62].

#### 3.6.5. Organ and End-Point Toxicity Prediction

ProTox-II online tool toxicity prediction test was utilized to investigate the safety profile of all compounds. This server categorized compounds into six toxicity classes (1–6), which projected the lethal dose (LD_50_) (mg/kg) and toxicity class of the identified ligands. Class one possesses lethal toxicity with an estimated lethal dosage (LD_50_) of 5, and class six demonstrates an LD_50_ > 5000, indicating the compound is less toxic [23].

### 3.7. Statistical Analysis

The data were expressed as mean ± standard deviation calculated using Microsoft Office Excel. The experiments were performed in triplicate, and the average mean was calculated. IC_50_ values were calculated using GraphPad Prism7 software.

## 4. Conclusions

In brief, the findings of the present investigation support the valuable anti-cancer and anti-inflammatory activity of myrrh; in addition, the wound healing properties could be attributed to its ability to modulate macrophage function. Using in vitro testing, our results revealed that myrrh had an anti-cancer effect against several cancer cell lines and an immunomodulatory effect with acceptable ADME properties. This could be used as a starting point to explore the potential biological properties of each active compound. Together, these results provide insights into the potential therapeutic activity of myrrh extract, which warrants further efficacy and safety investigations.

## Figures and Tables

**Table 1:** Characterization and identification of metabolites found in myrrh methanolic extract using high-performance liquid chromatography time-of-flight mass spectrometry (HPLC/DAD-QTOF MS/MS).

Peak No.	ESI-MS *Rt* (min)	Observed Mass (*m*/*z*)	Calculated Mass (*m*/*z*)	Error (ppm)	Molecular Formula	Key MS^E^ Fragment Ions (*m*/*z*)	Identification
A	6.13	288.148	288.136	−0.5	C_17_H_20_O_4_	[M-CH_3_]^+^ 273.113, [M-2CH_3_]^+^ 259.089, [M-3CH_3_]^+^ 243.118, and [M-CO_2_CH_3_]^+^ 246.118	4,5-Dihydrofuranodienone
B	6.35	231.155	231.131	−1.8	C_15_H_18_O_2_	[M-CH_3_]^+^ 215.107, [M-2CH_3_]^+^ 200.084, and [M-3CH_3_]^+^ 185.023	Furanodienone
C	6.78	313.157	313.149	−0.5	C_22_H_32_O_2_	[M-CH_3_]^+^ 297.222, [M-2CH_3_]^+^ 282.198, [M-3CH_3_]^+^ 267.175, [M-4CH_3_]^+^ 252.151, and [M-5CH_3_]^+^ 237.128,	Mansumbinone
D	7.25	269.133	269.129	−0.7	C_20_H_30_	[M-CH_3_]^+^ 258.235, [M-2CH_3_]^+^ 243.211, [M-3CH_3_]^+^ 228.188, [M-4CH_3_]^+^ 213.164, and [M-C_3_H_5_]^+^ 231.211, 41.039	Cembrene-A
E	6.93	404.421	404.417	−0.6	C_28_H_48_O	[M-CH_3_]^+^ 385.347, [M-2CH_3_]^+^ 370.324, [M-3CH_3_]^+^ 355.300, [M-4CH_3_]^+^ 340.277, [M-5CH_3_]^+^ 325.253, and [M-C_9_H_19_]^+^ 273.222, 127.149.	Campestan-3b-ol
F	7.70	301.158	301.147	−0.9	C_21_H_28_O_2_	[M-CH_3_]^+^ 301.217, [M-2CH_3_]^+^ 286.193, and [M-3CH_3_]^+^ 271.170.	Pregnadienes
G	8.08	253.133	253.125	−0.8	C_15_H_18_O_2_	[M-CH_3_]^+^ 238.131, [M-2CH_3_]^+^ 223.107, and [M-3CH_3_]^+^ 208.084.	Z,4Z-Furanodien-6-one
H	8.64	418.7695	416	−1.2	C_27_H_44_O_3_	[M-CH_3_]^+^ 374.266, [M-2CH_3_]^+^ 359.243, [M-3CH_3_]^+^ 355.300, [M-4CH_3_]^+^ 344.243, [M-5CH_3_]^+^ 329.219, and [M-C_8_H_17_]^+^ 273.222, 113.133.	Guggulsterols
I	9.66	331.2109	331.2097	−1.4	C_22_H_34_O_2_	[M-CH_3_]^+^ 315.232, [M-2CH_3_]^+^ 300.209, [M-3CH_3_]^+^ 285.185, and [M-C_2_H_3_O_2_]^+^ 271.243	3,4-Seco-mansumbinoic acid

**Table 2:** The IC_50_ values of myrrh using breast, colorectal, and leukemia cell lines and primary fibroblasts and normal blood cells.

Cancer Cell Line	IC_50_ ± SE	R2
Leukemia (HL60)	26.29 ± 7.09	0.957
Leukemia (K562)	88.27± 12.58	0.805
Breast Cancer (KAIMRC1)	95.73 ± 6.48	0.979
Breast Cancer (MDA-MB-231)	272.6 ± 6.561	0.976
Colorectal Cancer (HCT8)	150 ± 11.35	0.958
Colorectal Cancer (HCT116)	132.9 ± 4.90	0.988
Normal Blood Sample (N1)	27.59 ± 11.14	0.911
Normal Primary Fibroblasts (P1)	295.4 ± 15.98	0.927

**Table 3:** Prediction of biological activity of myrrh methanolic extracts.

Compound Number (Name)	Predicted Activity	P_a_	P_i_
1 (4,5-Dihydrofuranodienone)	Anti-inflammatory	0.390	0.101
	Anti-oxidant	0.324	0.019
	Wound-healing agent	-	-
	Free radical scavenger	0.284	0.034
	Anti-neoplastic	0.879	0.005
2 (Furanodienone)	Anti-inflammatory	0.449	0.073
	Anti-oxidant	0.319	0.020
	Wound-healing agent	-	-
	Free radical scavenger	0.255	0.044
	Anti-neoplastic	0.788	0.013
3 (Mansumbinone)	Anti-inflammatory	0.506	0.055
	Anti-oxidant	0.155	0.097
	Wound-healing agent	0.168	0.161
	Free radical scavenger	-	-
	Anti-neoplastic	0.814	0.010
4 (Campestan-3b-ol)	Anti-inflammatory	0.602	0.031
	Anti-oxidant	0.157	0.095
	Wound-healing agent	0.197	0.121
	Free radical scavenger	-	-
	Anti-neoplastic	0.824	0.009
5 (Cembrene-A)	Anti-inflammatory	0.497	0.058
	Anti-oxidant	0.181	0.068
	Wound-healing agent	0.416	0.020
	Free radical scavenger	-	-
	Anti-neoplastic	0.340	0.130
6 (Pregnadienes)	Anti-inflammatory	0.564	0.040
	Anti-oxidant	0.171	0.078
	Wound-healing agent	-	-
	Free radical scavenger	-	-
	Anti-neoplastic	0.888	0.005
7 (Z,4Z-Furanodien-6-one)	Anti-inflammatory	0.449	0.073
	Anti-oxidant	0.319	0.020
	Wound-healing agent	-	-
	Free radical scavenger	0.255	0.044
	Anti-neoplastic	0.788	0.013
8 (Guggulsterols)	Anti-inflammatory	0.559	0.041
	Anti-oxidant	0.192	0.060
	Wound-healing agent	0.192	0.127
	Free radical scavenger	-	-
	Anti-neoplastic	0.593	0.047
9 (3,4-Seco-mansumbinoic acid)	Anti-inflammatory	0.510	0.054
	Anti-oxidant	0.192	0.060
	Wound-healing agent	-	-
	Free radical scavenger	-	-
	Anti-neoplastic	0.735	0.020

P_a_: Probability of being active; P_i_: Probability of being inactive.

**Table 4:** Target prediction of crude myrrh resin using Molinspiration webserver.

Compounds Number (Name)	GPCR Ligand	Ion Channel Modulator	Kinase Inhibitor	Nuclear Receptor Ligand	Protease Inhibitor	Enzyme Inhibitor
Crude myrrh resin						
1 (4,5-Dihydrofuranodienone)	−0.20	−0.23	−0.77	0.04	−0.48	0.30
2 (Furanodienone)	−0.45	−0.42	−0.96	−0.27	−0.85	0.05
3 (Mansumbinone)	0.08	−0.22	−0.72	0.60	−0.20	0.46
4 (Campestan-3b-ol)	0.02	0.07	−0.51	0.38	−0.23	0.24
5 (Cembrene-A)	0.13	0.13	−0.46	0.78	0.08	0.56
6 (Pregnadienes)	−0.06	−0.09	−0.91	0.99	−0.23	0.50
7 (Z,4Z-Furanodien-6-one)	−0.45	−0.42	−0.96	−0.27	−0.85	0.05
8 (Guggulsterols)	0.20	0.08	−0.60	0.99	0.12	0.71
9 (3,4-Seco-mansumbinoic acid)	0.17	0.05	−0.58	0.73	−0.10	0.43

**Table 5:** Target prediction of crude myrrh resin using Swiss Target and SEA Search.

Compound Number (Name)	SEA Search			Swiss Target	
	Predicted Targets	*p* Value	MaxTC	Predicted Targets	Probability
1 (4,5-Dihydrofuranodienone)	Glucocorticoid receptor Androgen receptor	Inactive Inactive	Inactive Inactive	Cyclooxygenase-2 Glucocorticoid receptor Beta amyloid A4 protein Androgen receptor DNA topoisomerase I	NA NA NA 0.098 NA
2 (Furanodienone)	Glucocorticoid receptor Androgen receptor	Inactive Inactive	Inactive Inactive	Cyclooxygenase-2 Glucocorticoid receptor Beta amyloid A4 protein Androgen receptor DNA topoisomerase I	0.049 NA 0.049 NA NA
3 (Mansumbinone)	Glucocorticoid receptor Androgen Receptor	Inactive Inactive	Inactive Inactive	Cyclooxygenase-2 Glucocorticoid receptor Beta amyloid A4 protein Androgen Receptor DNA topoisomerase I	NA 0.102 NA 0.102 NA
4 (Campestan-3b-ol)	Glucocorticoid receptor Androgen receptor	Inactive Inactive	Inactive Inactive	Cyclooxygenase-2 Glucocorticoid receptor Beta amyloid A4 protein Androgen receptor DNA topoisomerase I	NA NA NA NA NA
5 (Cembrene-A)	Glucocorticoid receptor Androgen receptor	Inactive 4.441 × 10^−16^	Inactive 0.53	Cyclooxygenase-2 Glucocorticoid receptor Beta amyloid A4 protein Androgen receptor DNA topoisomerase I	NA NA NA 0.631 NA
6 (Pregnadienes)	Glucocorticoid receptor Androgen receptor	1.121 × 10^−12^ 1.121 × 10^−12^	1.00 1.00	Cyclooxygenase-2 Glucocorticoid receptor Beta amyloid A4 protein Androgen receptor DNA topoisomerase I	NA 1.0 NA 1.0 NA
7 (Z,4Z-Furanodien-6-one)	Glucocorticoid receptor Androgen receptor	Inactive Inactive	Inactive Inactive	Cyclooxygenase-2 Glucocorticoid receptor Beta amyloid A4 protein Androgen receptor DNA topoisomerase I	0.049 NA 0.049 NA NA
8 (Guggulsterols)	Glucocorticoid receptor Androgen receptor	1.868 × 10^−23^ 1.218 × 10^−19^	0.40 0.53	Cyclooxygenase-2 Glucocorticoid receptor Beta amyloid A4 protein Androgen Receptor DNA topoisomerase I	0.119 0.473 NA 0.376 NA
9 (3,4-Seco-mansumbinoic acid)	Glucocorticoid receptor Androgen Receptor	Inactive Inactive	Inactive Inactive	Cyclooxygenase-2 Glucocorticoid receptor Beta amyloid A4 protein Androgen receptor DNA topoisomerase I	0.105 0.105 NA 0.113 0.105

NA: not applicable.

**Table 6:** Predictions of the pharmacokinetics ADME properties of crude myrrh resins using SwissADME computational tool.

Compounds Number (Name)	Molecular Weight (g/mol	HB Donor	HB Acceptor	Log Po/w (WLOGP)	Log S (SILICO S-IT)	BBB Permeant	GI Absorption	Rule of Five (ROF)
Crude Myrrh Resin								
1 (4,5-Dihydrofuranodienone)	288.34	0	4	3.54	−4.23 Moderately soluble	Yes	High	Yes; 0 violation
2 (Furanodienone)	230.30	0	2	4.00	−4.40 Moderately soluble	Yes	High	Yes; 0 violation
3 (Mansumbinone)	312.49	0	1	5.71	−5.18 Moderately soluble	No	High	Yes; 0 violation
4 (Campestan-3b-ol)	272.47	0	0	6.76	−5.18 Moderately soluble	No	High	Yes; 0 violation
5 (Cembrene-A)	400.68	1	1	7.78	−6.17 Poorly soluble	No	Low	Yes; 0 violation
6 (Pregnadienes)	312.45	0	2	4.64	−4.58 Moderately soluble	Yes	High	Yes; 0 violation
7 (Z,4Z-Furanodien-6-one)	230.30	0	2	4.00	−4.40 Moderately soluble	Yes	High	Yes; 0 violation
8 (Guggulsterols)	432.64	3	4	4.65	−4.12 Moderately soluble	No	High	Yes; 0 violation
9 (3,4-Seco-mansumbinoic acid)	230.50	1	2	5.84	−4.44 Moderately soluble	Yes	High	Yes; 0 violation

**Table 7:** CYP enzyme inhibition profile for crude myrrh resin using SWISSADME Webserver.

Compound Number (Name)	CYP1A2	CYP2C19	CYP2C9	CYP2D6	CYP3A4
1 (4,5-Dihydrofuranodienone)	No	Yes	No	No	No
2 (Furanodienone)	Yes	No	No	No	No
3 (Mansumbinone)	No	Yes	Yes	No	No
4 (Campestan-3b-ol)	No	Yes	Yes	No	No
5 (Cembrene-A)	No	No	No	No	No
6 (Pregnadienes)	No	Yes	Yes	No	No
7 (Z,4Z-Furanodien-6-one)	Yes	No	No	No	No
8 (Guggulsterols)	No	No	No	No	No
9 (3,4-Seco-mansumbinoic acid)	Yes	No	No	No	No

**Table 8:** Toxicity profiles of myrrh bioactive compounds using ProTox-II online tool.

Compound Number (Name)	Oral Toxicity of Compounds		Prediction of Active Organ Toxicity/Toxicity Endpoints	Probability
	Predicted LD_50_ (mg/kg)	Predicted Toxicity Class		
1 (4,5-Dihydrofuranodienone)	590	4	Hepatotoxicity	0.61 (Inactive)
			Immunotoxicity	0.73 (Inactive)
			Carcinogenicity	0.52 (Inactive)
			Mutagenicity	0.65 (Inactive)
2 (Furanodienone)	116	3	Hepatotoxicity	0.71 (Inactive)
			Immunotoxicity	0.96 (Inactive)
			Carcinogenicity	0.58 (Inactive)
			Mutagenicity	0.84 (Inactive)
3 (Mansumbinone)	15,000	6	Hepatotoxicity	0.70 (Inactive)
			Immunotoxicity	0.73 (Inactive)
			Carcinogenicity	0.57 (Inactive)
			Mutagenicity	0.97 (Inactive)
4 (Campestan-3b-ol)	4400	5	Hepatotoxicity	0.77 (Inactive)
			Immunotoxicity	0.93 (Inactive)
			Carcinogenicity	0.66 (Inactive)
			Mutagenicity	0.87 (Inactive)
5 (Cembrene-A)	890	4	Hepatotoxicity	0.87 (Inactive)
			Immunotoxicity	0.99 (Active)
			Carcinogenicity	0.60 (Inactive)
			Mutagenicity	0.98 (Inactive)
6 (Pregnadienes)	2300	5	Hepatotoxicity	0.67 (Inactive)
			Immunotoxicity	0.98 (Active)
			Carcinogenicity	0.56 (Active)
			Mutagenicity	0.99 (Inactive)
7 (Z,4Z-Furanodien-6-one)	116	3	Hepatotoxicity	0.71 (Inactive)
			Immunotoxicity	0.96 (Inactive)
			Carcinogenicity	0.58 (Inactive)
			Mutagenicity	0.84 (Inactive)
8 (Guggulsterols)	5010	6	Hepatotoxicity	0.82 (Inactive)
			Immunotoxicity	0.98 (Active)
			Carcinogenicity	0.66 (Active)
			Mutagenicity	0.67 (Inactive)
9 (3,4-Seco-mansumbinoic acid)	11,800	2	Hepatotoxicity	0.51 (Active)
			Immunotoxicity	0.93 (Inactive)
			Carcinogenicity	0.67 (Inactive)
			Mutagenicity	0.88 (Inactive)

**Table 9:** Color code for the toxicity prediction test.

Class 1:	Fatal if swallowed (LD_50_ ≤ 5)
Class 2:	Fatal if swallowed (5 < LD_50_ ≤ 50)
Class 3:	Toxic if swallowed (50 < LD_50_ ≤ 300)
Class 4:	Harmful if swallowed (300 < LD_50_ ≤ 2000)
Class 5:	It may be harmful if swallowed (2000 < LD_50_ ≤ 5000)
Class 6:	Non-toxic (LD_50_ > 5000)

## Data Availability

Data are contained within the Article.
